# New Reactive Force Field for Simulations of MoS_2_ Crystallization

**DOI:** 10.1021/acs.jpcc.2c01075

**Published:** 2022-05-26

**Authors:** I. Ponomarev, T. Polcar, P. Nicolini

**Affiliations:** Department of Control Engineering, Faculty of Electrical Engineering, Czech Technical University in Prague, Technicka 2, 16627 Prague 6, Czech Republic

## Abstract

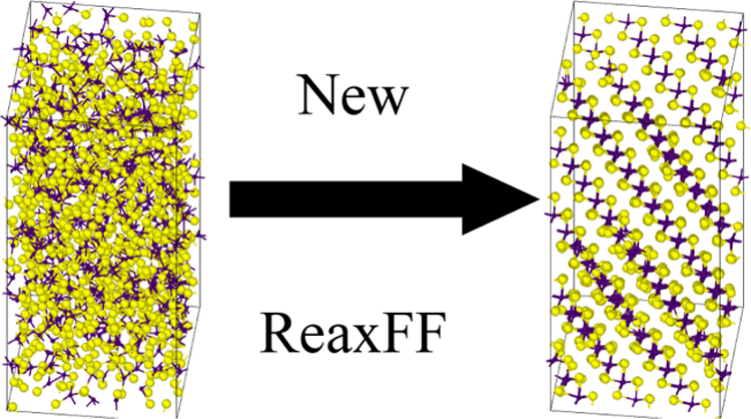

We present a new reactive force field (ReaxFF) parameter set for simulations of Mo–S structures. We compare our parameterization to the state-of-the-art ones in their performance against density functional theory (DFT) benchmarks and MoS_2_ crystallization simulations. Our new force field matches DFT data significantly better than any previously published force fields and provides a realistic layered MoS_2_ structure in crystallization simulations. It significantly improves the state-of-the-art force fields, which tend to crystallize in the experimentally unknown rock-salt MoS structure. Therefore, our new force field is a good candidate for further development and inclusion of other practically relevant elements, such as O, C, N, and H, which can be used to study the formation and tribological or catalytical properties of molybdenum disulfide.

## 1 Introduction

Molybdenum disulfide, MoS_2_, is a layered material from the transition metal dichalcogenide (TMD) family. This material is known from ancient times.^1^ The structure of the crystalline MoS_2_ comprises covalently bonded layers, held together by weak van der Waals interactions.^2^ The broad range of applications of the TMD-based materials includes tribological coatings^3−5^ and materials for electronics^6−8^ and catalysis.^9−12^

TMD thin films are often prepared via some deposition process, which often yields an amorphous material.^13^ Tribological applications rely on the natural tendency of TMDs to crystallize into a layered structure and form superlubricious sliding surfaces in the course of exploitation. Some other applications, however, require further treatment to crystallize the TMD film in a special way. For example, MoS_2_ coatings for flexible, stretchable photodetectors require very high crystallinity and, at the same time, relatively mild treatment conditions to avoid damage to the polymer substrate.^8^ Catalysis applications, on the other hand, would benefit from generating specific defects: sulfur vacancies for hydrogenation of CO_2_ into methanol,^14^ edge sites for oxygen reduction,^11^ or both for the hydrogen evolution reaction.^15^

Simulations at a large scale for long simulation times can be very useful to study the crystallization processes and provide insights for the successful tailoring of the treatment conditions to achieve specific goals. Several computational studies of MoS_2_ crystallization have been published in recent years, utilizing AIREBO^16−18^ and ReaxFF empirical potentials.^19,20^

ReaxFF^21^ is an empirical potential that aims to bridge the gap between very quick but not so reliable calculations using simple empirical potentials and accurate but prohibitively expensive density functional theory (DFT) calculations. Once a parameter set is properly developed, ReaxFF is a perfect tool to study chemical reactions and structural changes at a relatively large scale (tens of thousands of atoms for nanoseconds at least) at the DFT level of accuracy for molecules^21−23^ and solids.^24,25^ This scale should allow capturing various collective events happening in the course of a chemical reaction or phase transformation and provide deeper insights into the mechanisms of the reactions.

Several parameter sets have been developed for a Mo–S system. One of these was aimed at reproducing deformations of MoS_2_ sheets,^26^ one more was used to study MoO_3_ reacting with sulfur to form MoS_2_,^27,28^ and another was applied to studies of crystallization of a single layer of the Mo–S system at various stoichiometries and oxygen contents.^20^ However, as we show in our study, all state-of-the-art sets are unsuitable for exploring crystallization of MoS_2_ outside the single-layer setup.

While doing ReaxFF parameter development for a V–O system starting from Chenoweth et al.’s set,^23^ we found a force field parameterization that looked promising for further development of the Mo–S system. This force field produced layered structures closely resembling MoS_2_ layers. Here, we present the outcome of our efforts in the further development of this force field, a new Mo–S force field.

## 2 Methods

The ReaxFF force field^21^ computes the total energy of the system as a sum of several components: bond energies, including lone pair correction, over- and undercoordination penalties, valence angle strains, torsion energies, Coulombic energies, and a number of fixes for specific cases like allenes, C–O bond, or bonding in C_2_
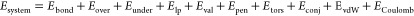
1

In our molecular dynamics (MD) simulations, we used ReaxFF as implemented in LAMMPS^29^ (reax/c package^30^). In melt-quench simulations, we used a time step of 0.5 fs, which we found to be a suitable choice in terms of energy conservation (see Figure S1). We applied the Polak–Ribiere version of the conjugate gradient (CG) algorithm (min_style cg in LAMMPS); stopping tolerances for energy and force were set to 10^–12^.

We performed reference DFT^31^ calculations within the Vienna Ab initio Simulation Package (VASP).^32,33^ We used the projector augmented wave method^34^ and approximated electron exchange and correlation within the Perdew–Burke–Ernzerhof^35,36^ generalized gradient approximation. The energy cutoff for the expansion of the wave function into the plane-wave basis was set to 500 eV. We used the DFT-D2 method of Grimme^37^ to account for van der Waals interactions. Stresses were converged to lower than 2 kbar; for amorphous models, forces were converged to 10 meV/Å and for crystalline models to 5 meV/Å. We did calculations both with and without a 3.5 eV U-correction;^38^ the results of the 2H–MoS_2_ structure optimization for our reference calculations (as well as for our new ReaxFF parameter set) are represented in Table 1.

**Table 1:** DFT- and ReaxFF-Computed 2H–MoS_2_ Structural Parameters

	cell parameters [Å]		
method	*a*	*c*	band gap [eV]
experimental	3.169^39^	12.324^39^	1.29^40^
DFT, GGA	3.189	12.401	1.01
DFT, GGA+*U*	3.199	12.407	1.03
ReaxFF, this work	3.209	11.901	

For the ReaxFF parameter development, we utilized a Monte-Carlo-like optimization scheme, inspired by ref (41), but not strictly following the same procedure. The training set originally included a set of experimental and hypothetical crystalline Mo*_x_*S*_y_* structures. The error of the parameter set was computed as the sum of the weighted (*w_i_*) squared differences between target properties *P*_*i*_^0^ computed with the reference method (DFT + *U*) and the same property *P_i_* computed within ReaxFF with the current parameter set
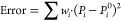


The properties considered include relative energies of the structures, atomic forces, cell parameters, bond distances, and bond angles. Starting from an educated guess (the V–O force field, derived from Chenoweth et al.,^23^ which produced VO_2_ layers resembling MoS_2_), at each step of the optimization process we applied small random changes to some of the relevant parameters (randomly chosen, up to a quarter of the total number) of the force field and computed the error for the new parameter set. During all steps, the best parameter set (i.e., the one with the smaller error) was updated and stored, together with its associated error. Then, if the error of the new parameter set was lower than the one at the previous step, the “move” was accepted; otherwise, the probability of the step to be accepted was defined as
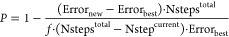
2where Nsteps^total^ is the total number of steps in the run, Nstep^current^ is the number of the current step, and *f* is a preselected factor, usually set equal to a value from 0.05 to 0.5. The f factor has the role of the maximum starting fictitious temperature in our Monte Carlo run. For example, if the factor is set at 0.05, the first step can only be accepted if the error of the step is smaller than 105% of the error of the starting set. The probability of making a successful step decreases with increasing the number of steps accepted, making it a “Monte Carlo melt-quench” simulation, while in ref (41), it is a “Monte Carlo annealing” procedure. With every unaccepted step, the maximum available change of the parameters was decreased 2-fold, and with every accepted step, the maximum available change of the parameters was increased 2-fold. After 10 unsuccessful attempts to move, the running set was replaced with the currently best parameter set to “restart” the optimization from the best place so far.

The Monte Carlo run stops upon reaching Nsteps^total^ steps or upon making more than 20 unsuccessful step attempts in a row.

**Figure 1:**
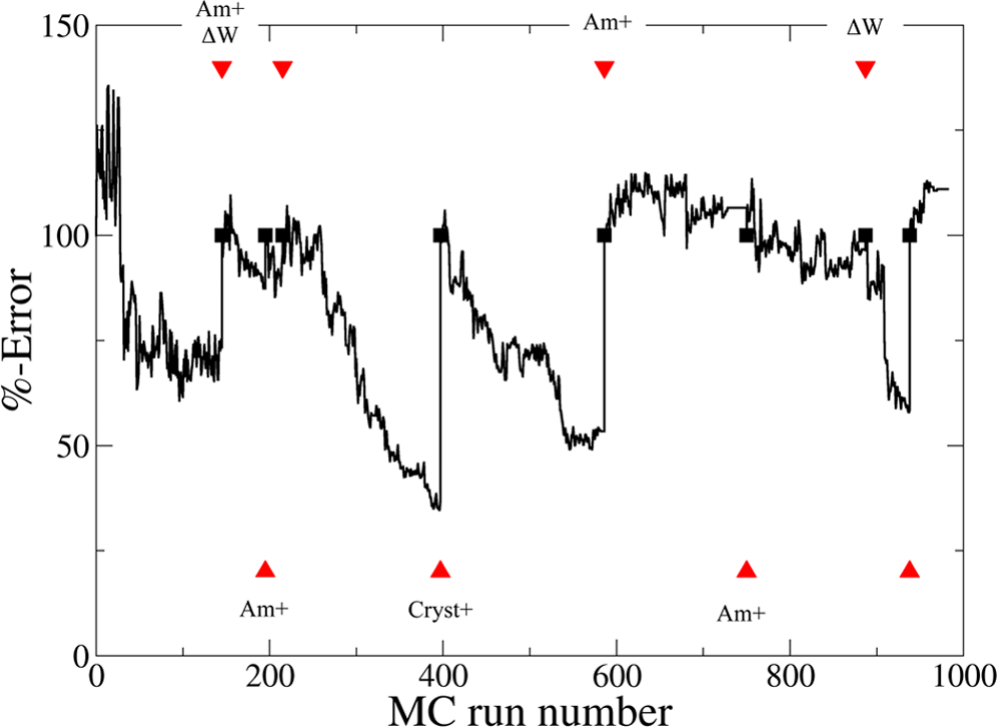
Evolution of the error during Monte Carlo runs. The error of the starting force field for each run is considered 100%. The start of the new run is marked with a black dot and a red triangle. “ΔW”, “Am+”, and “Cryst+” represent the new run mean changing of weights in the training set, adding amorphous structures to the training set, and adding crystalline structures to the training set, respectively.

During the Monte Carlo runs, we tested “currently best” force fields in terms of the ability to produce crystalline MoS_2_ in the melt-quench simulation. After some Monte Carlo runs, we expanded the training set with additional melt-quench-generated MoS_2_ models (melting at 5000 K, rapid cooling at 20 K/ps or quicker to try to preserve disorder in the models), which were further optimized within DFT. We also changed weights and added extra crystalline models in the course of optimization. Figure 1 shows the evolution of the error during our optimization runs.

## 3 Results and Discussion

### 3.1 Force Field Validation—Crystal Models

Table 1 shows the cell parameters of MoS_2_ computed within the DFT approach and using our new ReaxFF force field. The values of the DFT- and ReaxFF-computed lattice parameters are within 4% of error with respect to the experimental reference, which is a satisfactory level of correspondence.

**Figure 2:**
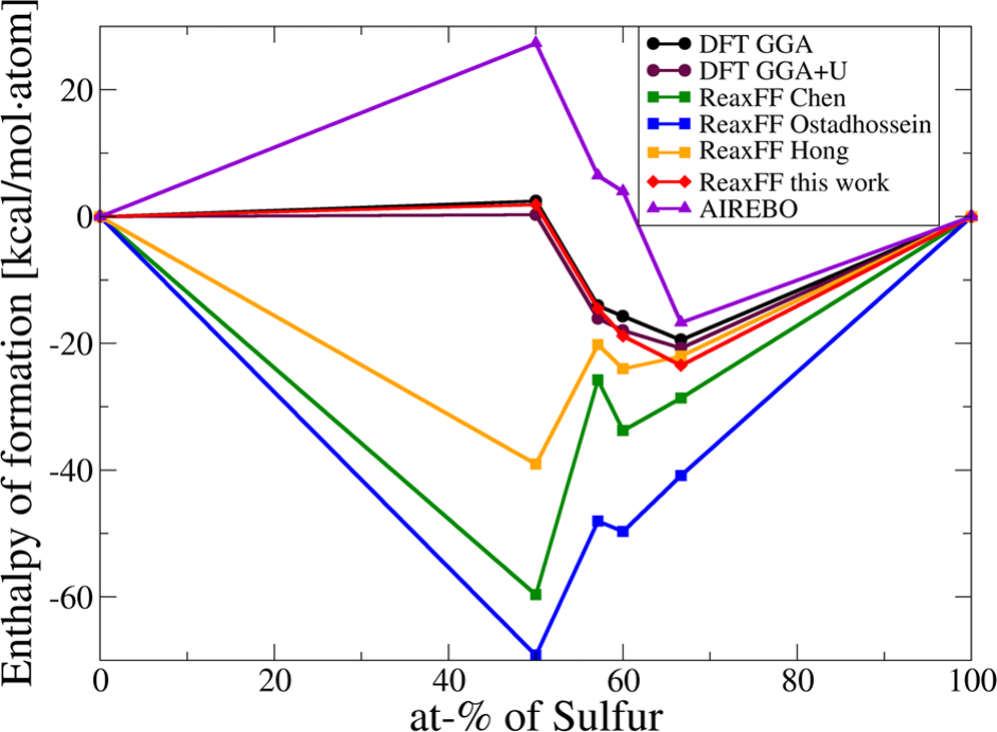
Convex hull diagram of the Mo*_x_*S*_y_* system, computed via DFT, AIREBO,^16^ and ReaxFF parameterizations: Chen,^20^ Ostadhossein,^26^ Hong,^28^ and our new force field parameters.

To further validate our new force field, we calculated a convex hull diagram for the Mo-–S system, as shown in Figure 2. On the diagram, we included the experimentally found phases listed in the Open Crystallography Database: Mo_3_S_4,_^42^ Mo_2_S_3_,^43^ and MoS_2._^39^ We also included the rock-salt-type MoS—this type of structure (distorted and defective) appeared in our attempts to crystallize MoS_2_ using state-of-the-art ReaxFF parameters outside the single-layer setup. As reference elemental structures, we used bcc-Mo^44^ and rhombohedral S_8_.^45^ We tested the force fields by Ostadhossein et al.,^26^ Chen et al.,^20^ and Hong et al.^28^ (and the AIREBO^16^ potential for the sake of completeness), as well as our new force field, vs reference DFT results.

Within DFT, both with and without U-correction, the hypothetical rock-salt-type MoS is unstable, even slightly endothermic. It is supposed to disproportionate to form bcc-Mo and Mo_3_S_4_. This is reasonable, since this phase is, to our best knowledge, unknown experimentally.

All three state-of-the-art ReaxFF parameterizations overestimate the stability of the rock-salt MoS phase, even to the extent that any other Mo*_x_*S*_y_* phase is unstable with respect to disproportionation into MoS and sulfur.

The AIREBO^16^ potential provides correct (compared to DFT) enthalpy of formation of MoS_2_, but the enthalpies of formation of every other crystalline phase are significantly underestimated.

The energies of Mo*_x_*S*_y_* crystals computed via our new parameterization are within 3 kcal/(mol·atom) with respect to DFT values, and the overall picture of the relative stabilities of the phases is the same as computed via the first-principles method.

In addition to defect-free bulk crystal structures, we also took a closer look at the energetics of stacking (adhesion/layer separation energy) and defects (Mo and S vacancies). We computed the energies of a relatively large single layer of MoS_2_ (64 formula units, 192 atoms in total) and the same layer with Mo and S vacancies. We defined layer separation energy as half of the energy difference (per atom) between a cell consisting of two layers of MoS_2_ in a bulk arrangement and when the layers are not interacting. We defined vacancy energy as the difference between the enthalpies of formation (from Mo and S) of the single layer of MoS_2_ and the same layer with the vacancy. The results are reported in Table 2.

**Table 2:** Energies of Vacancy Formation and Adhesion Energy in 2H–MoS_2_

property	DFT, GGA+*U* [kcal/(mol·atom)]	ReaxFF, this work [kcal/(mol·atom)]
layer separation energy	0.59	0.77
Mo vacancy	0.74	0.60
S vacancy	0.23	0.05

We see a good correspondence between DFT and ReaxFF values for both adhesion energy and energies of vacancy formation. It is worth noting that the intrinsic accuracy of DFT is estimated to be about 0.5 kcal/(mol·atom),^46^ further underlying the agreement.

**Figure 3:**
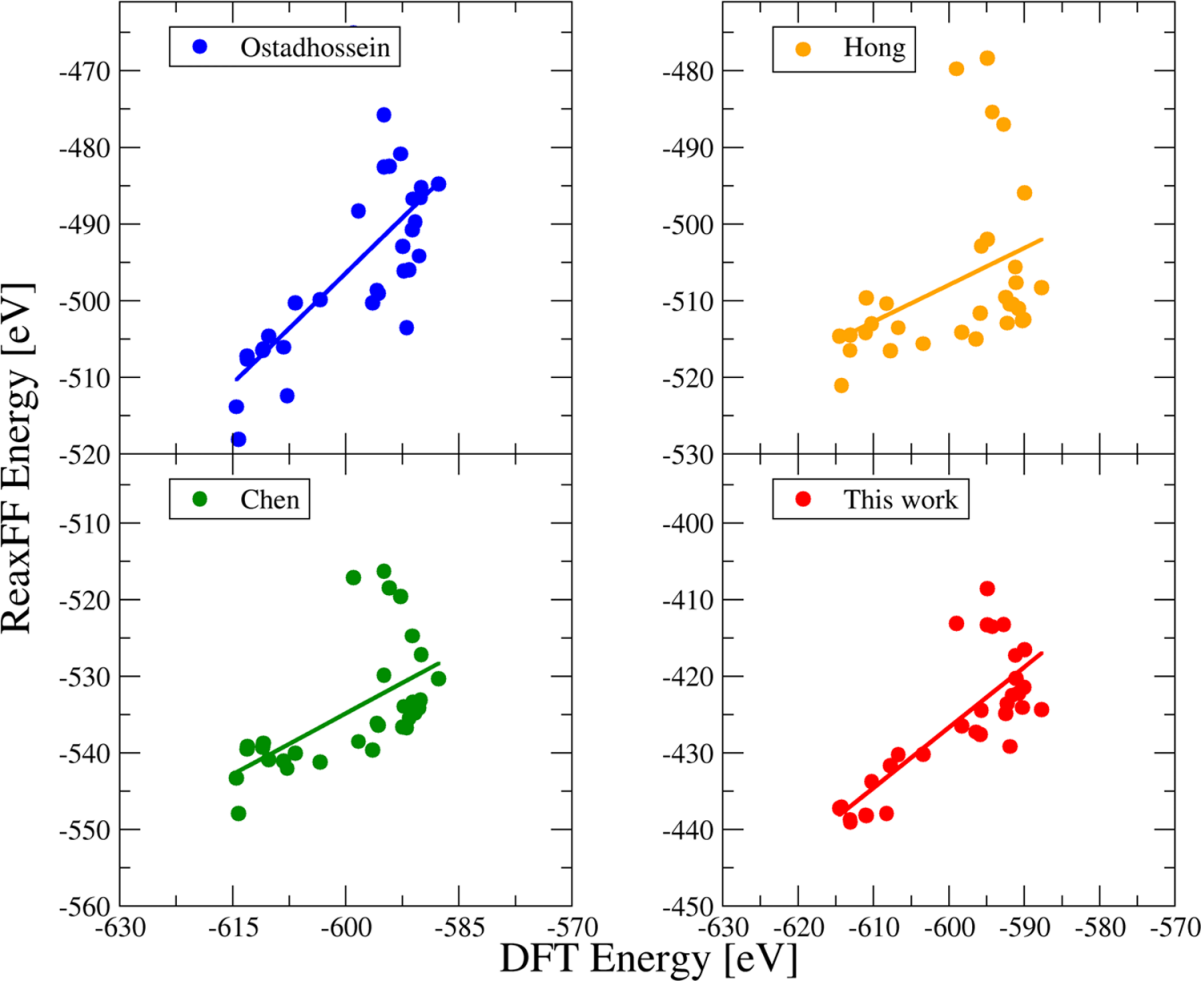
Energies of amorphous MoS_2_ models computed via DFT and various ReaxFF parameter sets: Ostadhossein,^26^ Hong,^28^ Chen,^20^ and the new force field.

We performed another test to assess the accuracy of the presented force field. As done in one of our previous works,^24^ we generated several amorphous models of MoS_2_ (density 5.06 g/cm^3^, cubic cell, 105 atoms) via melt-quench simulations (rapid quenching from 5000 K to 0 K, 20 K/ps and faster) within ReaxFF (the final parameter set as well as the number of “intermediate” force fields that were obtained at different stages of the optimization process). We further optimized the final configurations using DFT, and we calculated the total energies of the models using DFT and within different ReaxFF parameterizations. The results are represented in Figure 3 and Table 3.

**Table 3:** Parameters of the Linear Fits for the DFT vs ReaxFF Energy Relations for MoS_2_ Amorphous Models

force field	slope	*R*^2^ value
Ostadhossein^26^	1.0 ± 0.2	0.51
Hong^28^	0.5 ± 0.2	0.15
Chen^20^	0.5 ± 0.1	0.35
this work	0.8 ± 0.1	0.63

We see a better correspondence in the DFT and ReaxFF energies of MoS_2_ amorphous models for the ReaxFF parameter set presented in this study: the *R*^2^ value of the fit is the best one among the explored force fields.

Some extra tests of the parameter sets are represented in the Supporting Information (Figures S2 and S3).

### 3.2 Simulated Crystallization of Amorphous MoS_2_—Confined Environment

First, we attempted to crystallize a relatively small cell, which is an orthogonalized MoS_2_ supercell comprising 216 atoms with starting sizes of 18.97 Å x 16.43 Å x 11.16 Å. The cell is nonperiodic in the *z*-direction with Lennard-Jones walls on the edges.

**Figure 4:**
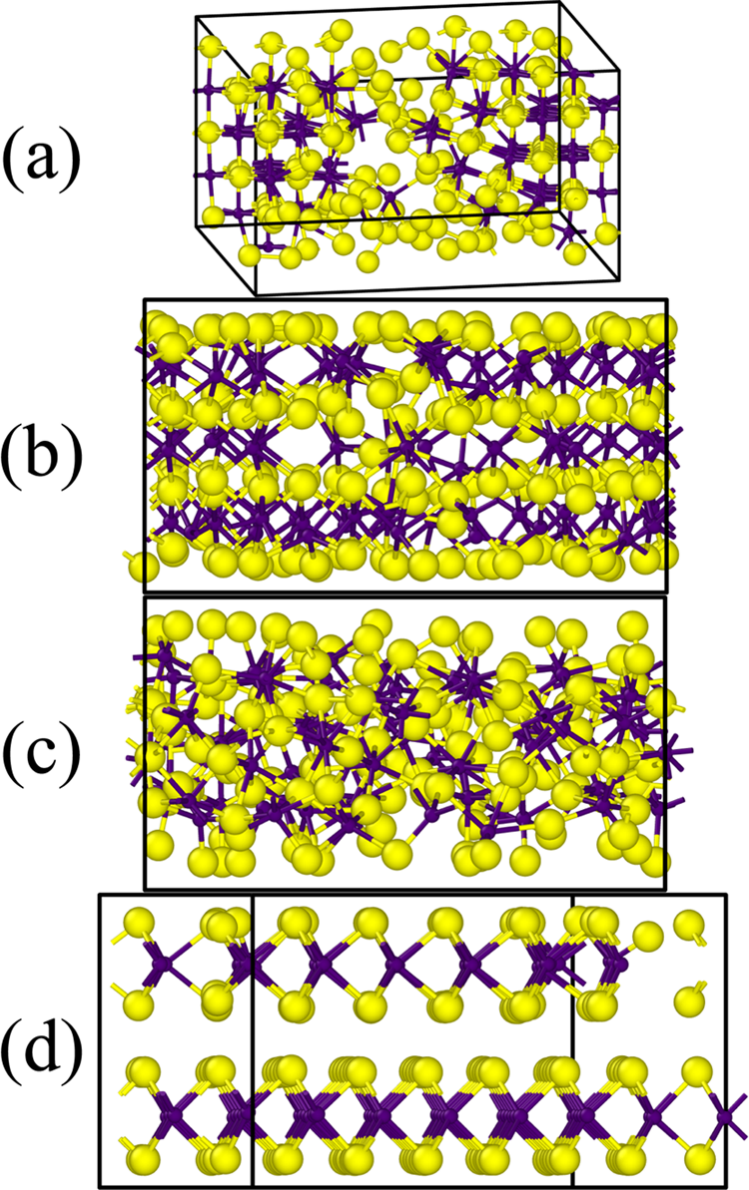
Final structures of the melt-quench simulations for 216-atom models of MoS_2_ with different ReaxFF parameterizations: Ostadhossein^26^ (a), Hong^28^ (b), Chen,^20^ (c) and our new ReaxFF (d) parameter sets. The view is generally along the *y*-axis; a slight rotation in panels (a) and (d) is added for a better representation of the crystalline phase.

**Figure 5:**
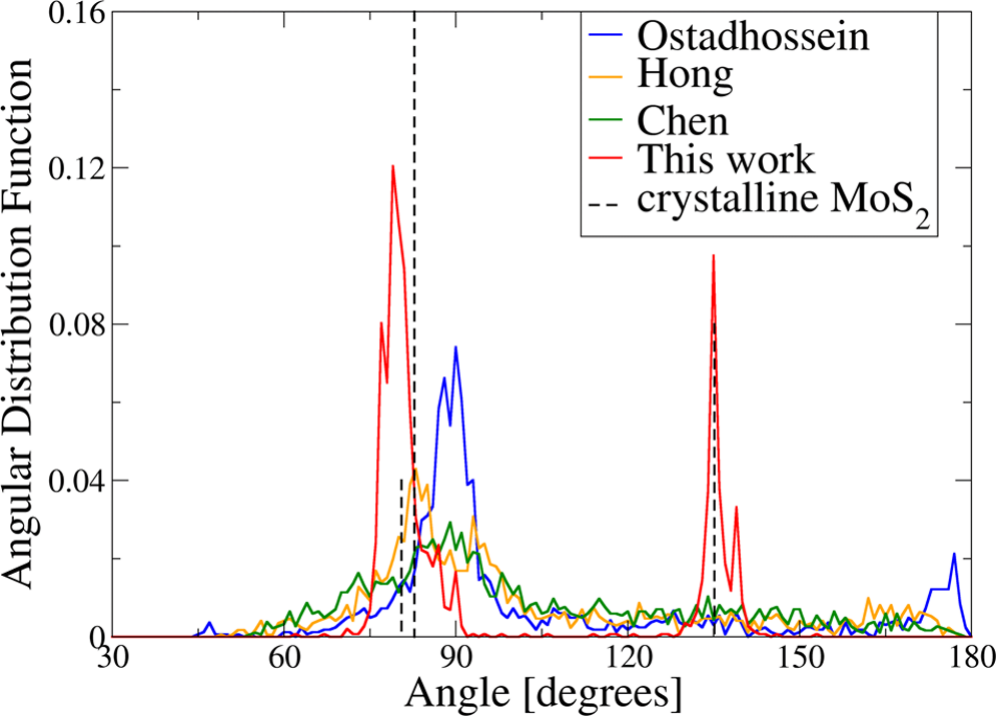
Angular distribution functions of the 216-atom models of MoS_2_ generated via melt-quenching with various ReaxFF parameterizations. Dashed lines represent the characteristic angles of DFT-optimized crystalline 2H-MoS_2_.

Previous studies explored the crystallization of MoS_2_ in a single-layer setup,^17−20^ where a single layer of MoS_2_ was sandwiched between MoS_2_ layers or layers of some other atoms. In our setup, we have a two-layer cell with van der Waals walls perpendicular to the *z*-direction on the top and the bottom of the cell. We optimized the initial orthogonalized MoS_2_ experimental structure to adjust the cell parameters for the parameter set in use, then heated the system from 0 K to 5000 K within 5 ps, melted the system at 5000 K for 50 ps, and then quenched it at a rate of 10 K/ps to 0 K. The outcomes in terms of structures and angular distribution functions are shown in Figures 4 and 5. Radial distribution functions are represented in the Supporting Information (Figure S4).

All state-of-the-art force fields do not crystallize the 2H–MoS_2_ structure.

The Ostadhossein force field crystallizes a structure resembling rock-salt-type MoS, though somewhat imperfect due to the system stoichiometry and box size constraints: some Mo atomic positions are not occupied, and octahedra might be distorted. The characteristic angle peaks are at 90° and at around 180°, and the MoS_2_ peak at 135° is missing.

The outcome of the simulation with the Chen force field is more chaotic and irregular, which is also pronounced in the angular distribution. The angular distribution function exhibits a very wide peak, centered at about 90° and tailing into the wide angles range. The characteristic peak at 135° is missing. Mo atoms are mostly 6-coordinated, while S atoms exhibit coordination numbers from 1 to 6, with “bulk” S atoms (those further away from the Lennard-Jones walls) being mostly 5- and 6-coordinated and “surface” S atoms (those closer to the Lennard-Jones walls) being 1- or 2-coordinated.

The final structure within the Hong force field is the one most resembling 2H–MoS_2_; however, some Mo atoms occupy interlayer positions instead of the proper sites within the layers, which is why the resulting structure lacks a pronounced characteristic peak of the crystalline MoS_2_ at 135°.

The outcome is not surprising, considering the convex hull diagrams: state-of-the-art force fields are heavily favoring the rock-salt structure (in some cases—with distortions), and from the convex hull point of view they are supposed to crystallize cubic MoS and sulfur. It is also not that surprising that in a single-layer setup, the simulation yields structures resembling MoS_2_: sulfur atoms tend to stick to van der Waals walls and be in the outer part of the cell, and the rest of the structure assembles to be as close to rock-salt MoS as possible, which is, in the end, very close to a MoS_2_ layer.

Our new force field yields slightly defective 2H–MoS_2_ layers: peak positions of the angular distribution function correspond well with those of crystalline MoS_2_. The layers are slightly rotated with respect to the expected direction of crystallization, which causes the defects.

### 3.3 Simulated Crystallization of Amorphous MoS_2_—Periodic Cell

Our simulation setup for a more extensive scale case is a random arrangement of 512 Mo and 1024 S atoms in a ratio of 1:2 at a density of crystalline MoS_2_ of 5.06 g/cm^3^. The cell is periodic and the dimensions are approximately 22 Å x 25 Å x 48 Å (with *x* and *y* dimensions of the cell being tailored to the specific force field in use). We heated the system to 5000 K within 50 ps, melted it at 5000 K for 50 ps, and then cooled it to 2000 K at 10 K/ps and held the system at 2000 K for 100 ps. We further quenched the system to 0 K at 10 K/ps.

**Figure 6:**
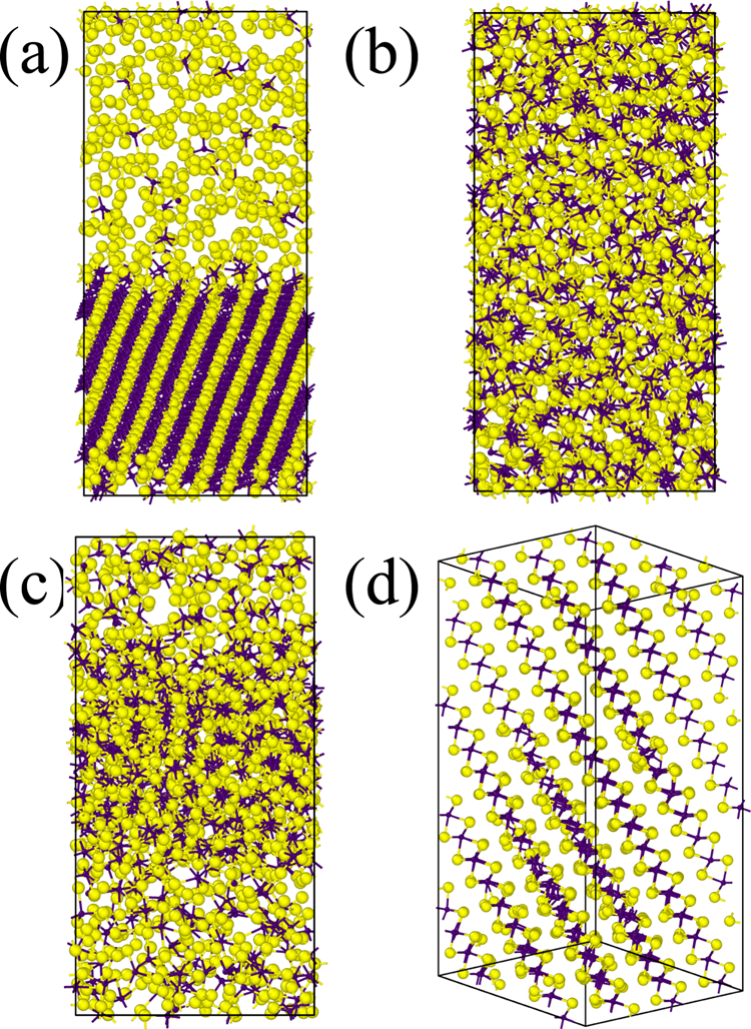
Final structures of the melt-quench simulations of 1536-atom models of MoS_2_ within different ReaxFF parameterizations: Ostadhossein^26^ (a), Hong^28^ (b), Chen,^20^ (c) and the new parameter set (d).

**Figure 7:**
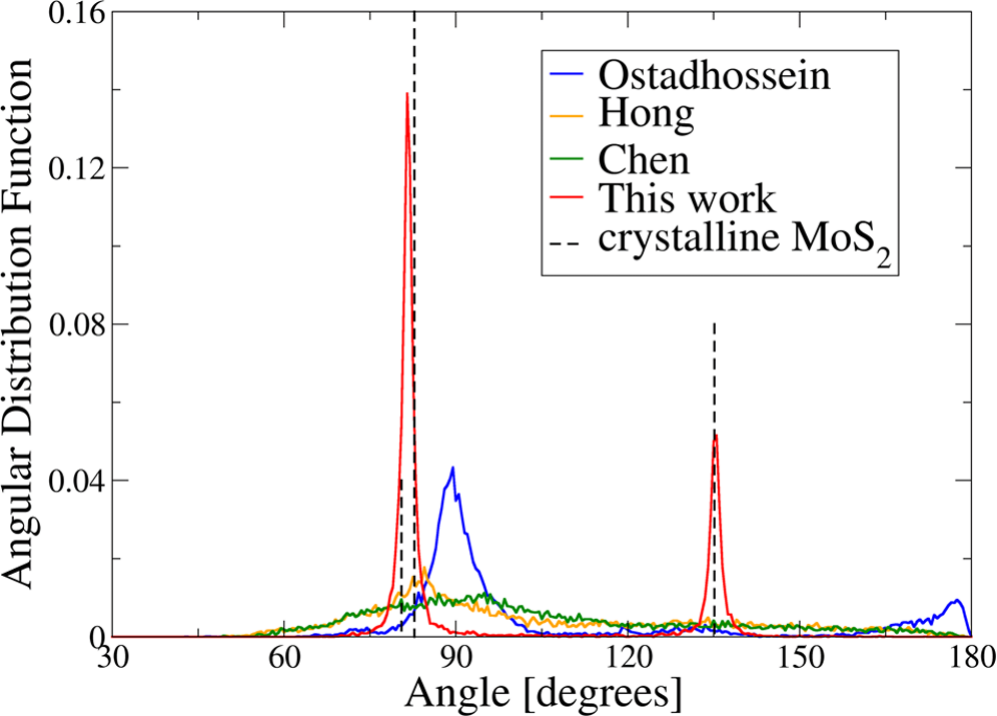
Angular distribution functions of the final structures of melt-quench simulations of 1536-atom models of MoS_2_ within different ReaxFF parameterizations. Dashed lines represent the characteristic angles of DFT-optimized crystalline 2H–MoS_2_.

We performed the simulations using the same four ReaxFF parameter sets. Figures 6 and 7 shows the outcomes of the simulations in terms of structures and angular distribution functions. Radial distribution functions are represented in the Supporting Information (Figure S5).

The outcome of the simulation with the Ostadhossein force field is quite similar to the previous case of the small constrained cell: a crystalline rock-salt MoS phase and a sulfur phase with a handful of Mo atoms. The angular distribution function has a pronounced peak centered around 90° and a peak near 180°, characteristic of the rock-salt-type structure.

For the Chen ReaxFF parameter set, the outcome is amorphous. Separation into Mo-rich and Mo-poor phases is not as pronounced as in the case of the Ostadhossein force field. The angular distribution function is centered at around 90° and very broad, without characteristic peaks of the crystalline MoS_2_.

The simulation with the Hong ReaxFF parameters also yielded amorphous MoS_2_, but, in this case, there is no visible phase separation. The angular distribution function exhibits a relatively pronounced peak at about 82°, which matches the one for 2H–MoS_2_; however, the characteristic peak at 135° is missing.

Our new ReaxFF parameter set yields almost perfect 2H–MoS_2_, which is visible both via the eye-ball test and on the angular distribution function: the structure is comprised of 2H–MoS_2_ layers and the peaks on the angular distribution function match the characteristic ones for the crystal.

## 4 Conclusions

We presented a new ReaxFF parameterization for the Mo–S system, which originated from working on the V–O ReaxFF parameter set. We compared it to the state-of-the-art Mo-S parameter sets^20,26,28^ in terms of relative stabilities of Mo*_x_*S*_y_* phases, energies of amorphous MoS_2_ models, and the ability to yield MoS_2_ crystallization in melt-quench simulations for more than one layer of MoS_2_ and without applying extremely high pressures and temperatures.

Our new ReaxFF parameterization matches DFT data in terms of the stability of crystalline phases, unlike the state-of-the-art parameter sets that tend to favor the hypothetical rock-salt MoS phase.

The superiority of our new parameter set is especially pronounced in the simulated crystallization of MoS_2_ outside the single-layer setup. The state-of-the-art parameterizations yield phases resembling rock-salt MoS; in the constrained single-layer setup with 1:2 stoichiometry, this indeed leads to the formation of the MoS_2_ layer, but if more freedom is allowed in the *z*-direction, it leads to phase separation into a cubic MoS and a sulfur phase. Our new ReaxFF force field, however, tends to produce layered MoS_2_. This tendency is not limited to small cells but also holds up for a more extensive system. We encourage the researchers to use this parameter set as the new state-of-the-art ReaxFF for future studies of the Mo–S systems.

Our new ReaxFF parameter set is a good starting point for further advancements, both in terms of improving Mo–S interaction description and adding extra elements (C, O, H, N) to broaden the scope of materials, the development of which can be supported with accurate and fast calculations.

## Supporting Information Available

Energy conservation in NVE simulations for amorphous MoS_2_, equation of state-like curves for crystalline MoS_2_, melting and quenching of MoS_2_, RDFs of MoS_2_ prepared via melt-quench (PDF)
Force field file, force field optimization setup (including python scripts, LAMMPS scripts, short description in “README” file and structures from the training set in LAMMPS input format) (ZIP)
