# Supplementary material for: New Reactive Force Field for Simulations of MoS2 Crystallization
Source: J Phys Chem C Nanomater Interfaces. 2022 May 26;126(22):9475–81. doi: 10.1021/acs.jpcc.2c01075 (PMC9189924; doi:10.1021/acs.jpcc.2c01075)
Supplement: Supplementary file 1 — jp2c01075_si_001.pdf [file jp2c01075_si_001.pdf]

A New Reactive Force Field for Simulations of MoS_2 crystallization

I. Ponomarev*, T. Polcar, P. Nicolini

Department of Control Engineering, Faculty of Electrical Engineering, Czech Technical University in Prague, Technicka 2, Prague 6, 16627, Czech Republic

* ponomili@fel.cvut.cz

Abstract

We present a new Reactive Force Field (ReaxFF) parameter set for simulations of Mo-S structures. We compare our parameterization to the state-of-the-art ones in their performance against DFT benchmarks and MoS_2 crystallization simulations. Our new force field matches DFT data significantly better than any previously published force fields and provides a realistic layered MoS_2 structure in crystallization simulations. It significantly improves the state-of-the-art force fields, which tend to crystallize in the experimentally unknown rock salt MoS structure.

Therefore, our new force field is a good candidate for further development and inclusion of other practically relevant elements, such as O, C, N and H, which can be used to study the formation and tribological or catalytical properties of molybdenum disulfide.

Keywords: molybdenum disulfide, crystallization, molecular dynamics simulations, ReaxFF, phase transition, amorphous coatings

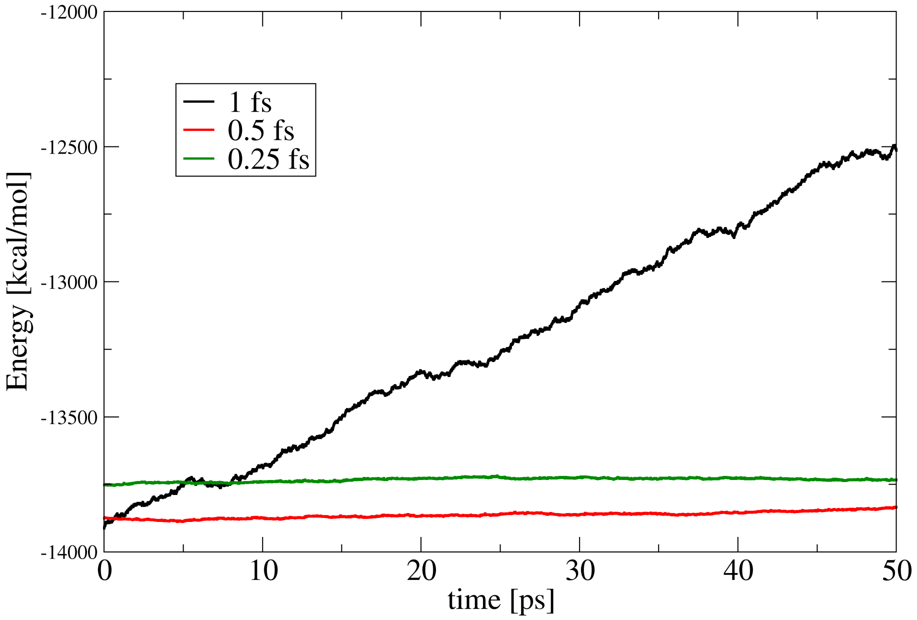

Figure S1. Energy conservation in Mo-S system within our new ReaxFF parameter set: NVE simulation with the initial temperature of 5000 K for the 120-atom amorphous MoS_2 model, preliminarily equilibrated at 2000 K.

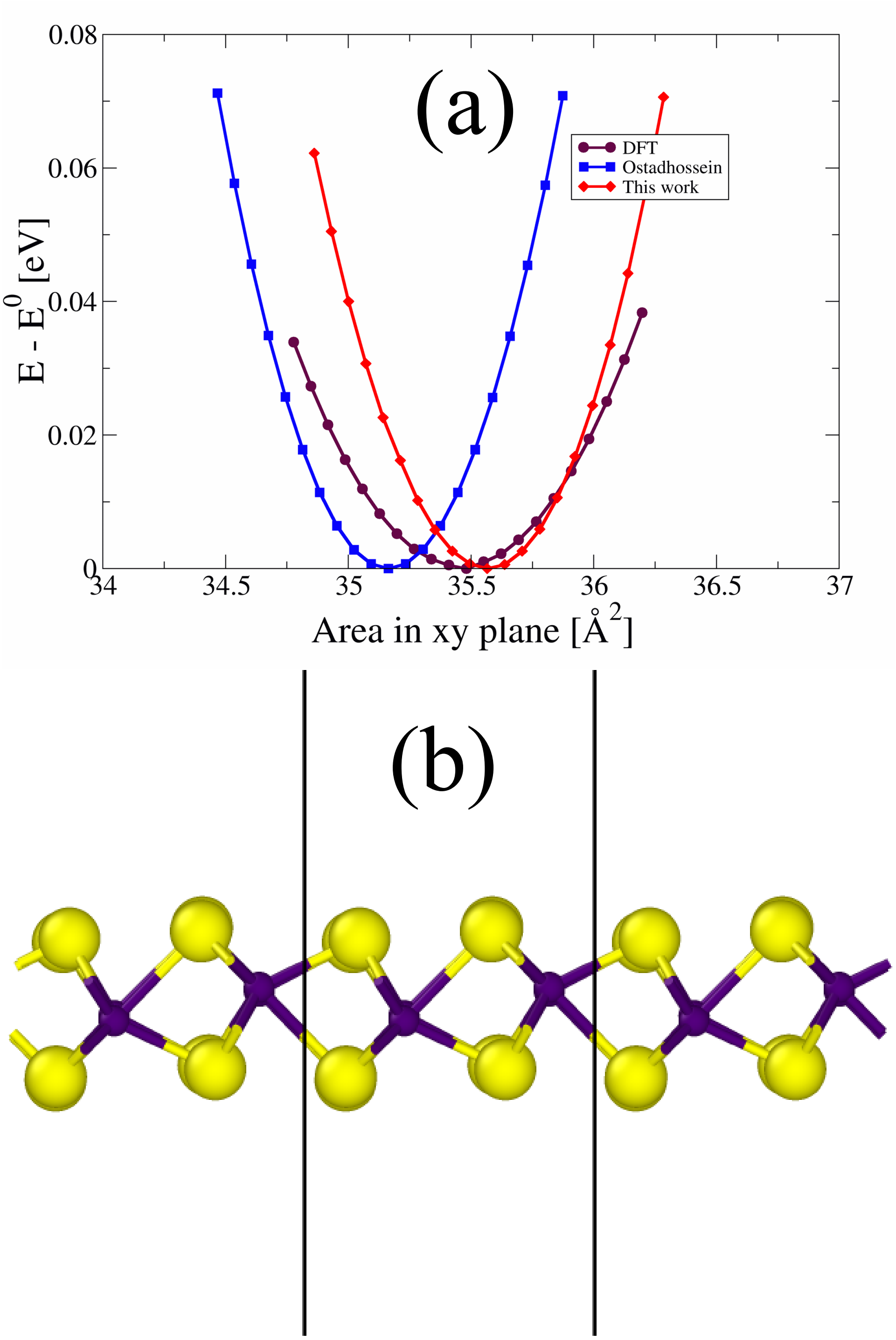

Figure S2. (a) energy vs. area in xy-plane curves for an orthogonalized layer of crystalline MoS_2 comprising 12 atoms, computed via DFT, Ostadhossein ReaxFF parameter set and our new ReaxFF parameter set. Hong and Chen parameter sets quickly yielded distortions of MoS_2 layer, diplayed in panel (b). Those distortions significantly reduced the total energy of the system within those force fields.

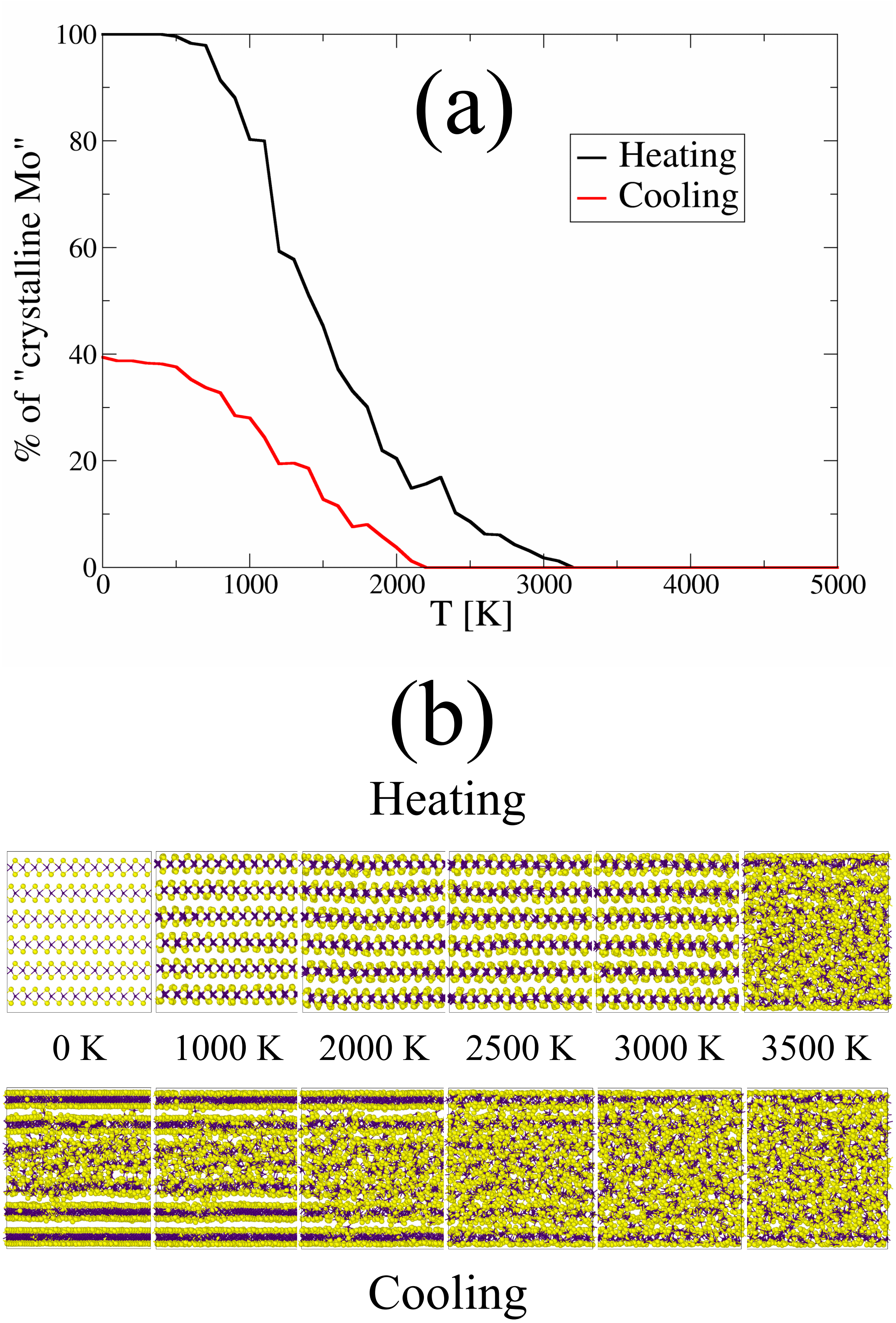

Figure S3. Melting and quenching simulations of MoS_2 model comprising 2160 atoms. The top and the bottom of the model in z-direction were Lennard-Jones van der Waals walls. The model was

heated to 5000 K, held at 5000 K for 100 ps and cooled to 0 K with the rate5 K/ps. We defined "Crystalline Mo" as Mo atoms, surrounded by 6 S atoms, that are 3-coordinated. (a) % of crystalline Mo atoms vs. temperature (b) snapshots of the structure during the simulation.

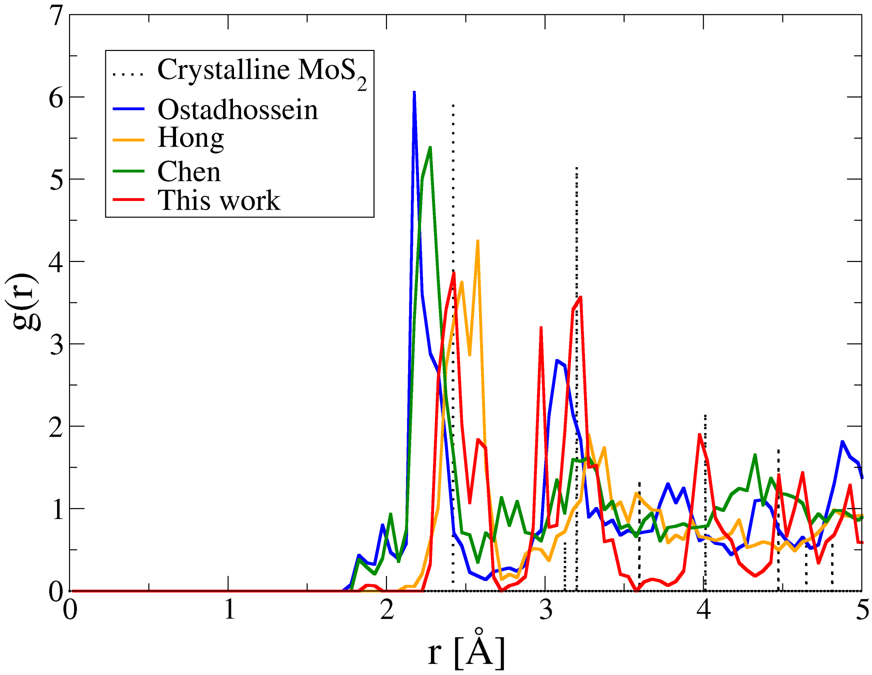

Figure S4. Angular distribution functions of the 216-atom models of MoS_2 generated via melt-quench with various ReaxFF parameterizations. Dashed lines represent the characteristic peaks of DFT-optimized crystalline 2H-MoS_2.

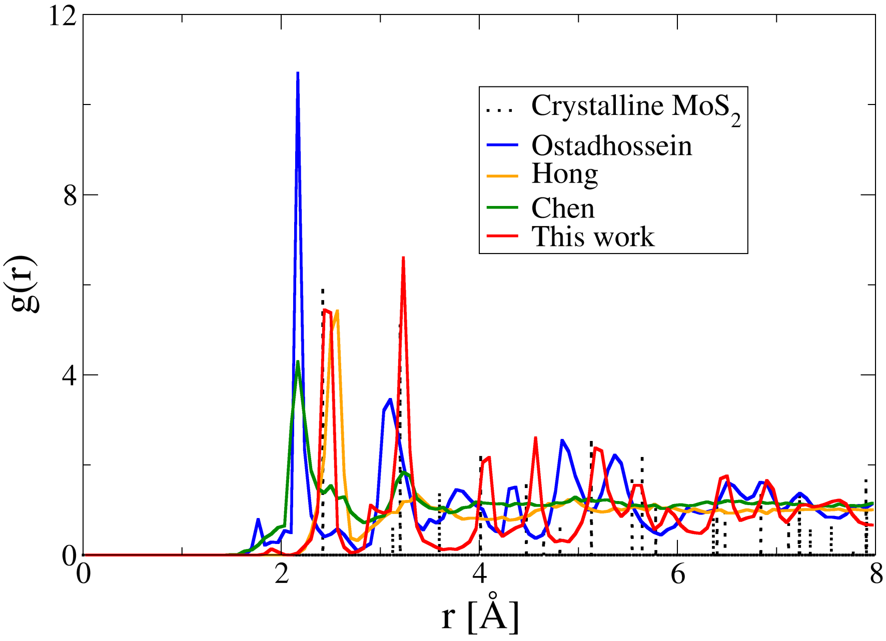

Figure S5. Angular distribution functions of the 1536-atom models of MoS_2 generated via melt-quench with various ReaxFF parameterizations. Dashed lines represent the characteristic peaks of DFT-optimized crystalline 2H-MoS_2.
